# An Ultrasensitive Laser-Induced Graphene Electrode-Based Triboelectric Sensor Utilizing Trapped Air as Effective Dielectric Layer

**DOI:** 10.3390/polym16010026

**Published:** 2023-12-20

**Authors:** Tapas Kamilya, Doohyun Han, Jaehee Shin, Soongeun Kwon, Jinhyoung Park

**Affiliations:** 1 Department of Mechatronics Engineering, Korea University of Technology & Education, 600, Chungjeol-ro, Byeongcheon-myeon, Dongnam-gu, Cheonan-si 31253, Republic of Korea; tkkt@koreatech.ac.kr (T.K.); hdh9903@koreatech.ac.kr (D.H.); wogmlchs@koreatech.ac.kr (J.S.); 2 Nano-Convergence Manufacturing Systems Research Division, Korea Institute of Machinery & Materials, 156, Gajeongbuk-ro, Yuseong-gu, Daejeon 34103, Republic of Korea

**Keywords:** LIG, air-trapped pad, ATPS, silicone rubber, PI

## Abstract

Air, a widely recognized dielectric material, is employed as a dielectric layer in this study. We present a triboelectric sensor with a laser-induced graphene (LIG) electrode and an air-trapped pad using silicone rubber (SR). A very thin device with a thickness of 1 mm and an effective gap for contact–separation between the films of silicone rubber and polyimide (PI) of 0.6 mm makes the device extremely highly sensitive for very low amplitudes of pressure. The fabrication of LIG as an electrode material on the surface of PI is the key reason for the fabrication of the thin sensor. In this study, we showed that the fabricated air-trapped padded sensor (ATPS) has the capability to generate an output voltage of ~32 V, a short-circuit current of 1.2 µA, and attain a maximum power density of 139.8 mW m^−2^. The performance of the ATPS was compared with a replicated device having a hole on the pad, allowing air to pass through during contact–separation. The observed degradation in the electrical output suggests that the trapped air in the pad plays a crucial role in enhancing the output voltage. Therefore, the ATPS emerges as an ultra-sensitive sensor for healthcare sensing applications.

## 1. Introduction

Self-powered sensors are currently an emerging field of research, and researchers are showing a growing interest in triboelectric sensors. Because of their versatility and the availability of a range of materials, triboelectric nanogenerators (TENGs) occupy a significant position in self-powered sensing applications [1,2,3,4]. Typical sensing systems possess some considerable drawbacks when utilized in real-time applications, including material selectivity, stability, complex circuitry, bulky sizes and high costs [5,6,7]. The invention of a TENG by the group led by Z. L. Wang presented a unique contribution in the development of self-powered sensing systems [8]. The TENG uses the technology of scavenging electrical energy via the production of a static charge caused by the friction of the surfaces of two active tribo layers; moreover, through an induction effect that occurs when charges are produced on the surfaces of the electrodes, they become dynamic and produce an electrical output [8,9,10,11]. The TENG is capable of scavenging electrical energy from mechanical energy, but, thus far, it has been observed that the output power is very low in TENGs [12,13,14]. Therefore, in parallel to the research seeking to enhance the output power, increasing research has been conducted on high-voltage applications and self-powered sensing applications using TENGs [15,16,17,18,19]. A range of versatile self-powered triboelectric sensors have been designed to date, where the sensing capabilities have been shaped through device engineering, introducing new materials, preparing composite materials and using piezo materials for hybrid nanogenerators [20,21,22,23]. A high-dielectric-constant material is a desirable choice for the development of a highly efficient TENG, as a high dielectric constant helps to produce a larger number of triboelectric charges due to triboelectrification [24,25]. Air possesses a certain dielectric constant, and, in most cases, the effects of charged air are ignored when developing TENGs [26,27]. The charged air is passed on in each contact–separation process. The flow of charged air diminishes the output performance of TENGs [26,27]. Herein, we report a triboelectric pressure sensor using an air-trapped pad with a laser-induced graphene (LIG) electrode. LIG has enabled breakthroughs in research and has attracted attention in recent years in the field of TENGs due to its excellent output performance and multiple real-time self-powered sensing applications [14,28,29,30]. We have seen a great deal of research on electrode materials for TENGs, where the very costly and high-end deposition or growth of electrode materials has been reported, such as the vacuum deposition technique and chemical vapor deposition (CVD). On the other hand, metal adhesion on PI films is poor due to the effect of the adhesive material [31,32]. The formation of LIG on the surface of a good triboelectric material provides an alternative durable material in the field of TENGs as it possesses a highly conductive electrode with a high-end tribo nature [14]. We have fabricated LIG using a CO_2_ laser on a polyimide (PI) film, as reported previously [14]. In this report, we also describe our research using a PI film where a CO_2_ laser was irradiated on one of its surfaces and we developed an array of electrodes. The advantage of using a PI film to produce LIG for a TENG is that the film of one material acts as both the triboelectric layer and conducting electrode, respectively. The PI layer acts as a good tribo-negative material and the LIG acts as a conducting electrode, which makes it attractive for real-time triboelectric sensing applications. In our study, we have developed a mask-generated air-trapped circular pad using silicone rubber. The flat surface of the PI film and the mask-generated padded film of silicone rubber are attached together using tape in such a way that prevents air leaks. The air-trapped padded area of 1.13 cm^2^ with a height of 0.6 mm is the key characteristic of our device air-trapped padded sensor (ATPS). A light touch on the air-trapped pad can cause contact–separation between the silicone rubber (SR) and PI film and produces triboelectrification and, hence, an electric output voltage. The device engineering along with the use of trapped air as a dielectric medium makes the ATPS highly sensitive over very low amplitudes under a range of pressure levels. We show how the trapped air impacts the sensitivity of the device by comparing the voltage output when the air in the pad is passed in each stage of contact and separation. A device size of 20 × 15 × 1 mm^3^, having an air-trapped padded area of 1.13 cm^2^, delivers a peak-to-peak output voltage of 32 V, a current of 1.2 µA and maximum power of 15.8 µW. We demonstrate that our device is potentially suitable for real-time multipurpose healthcare sensing applications.

## 2. Materials and Methods

### 2.1. Preparation of LIG Film Using PI Film

**Figure 1:**
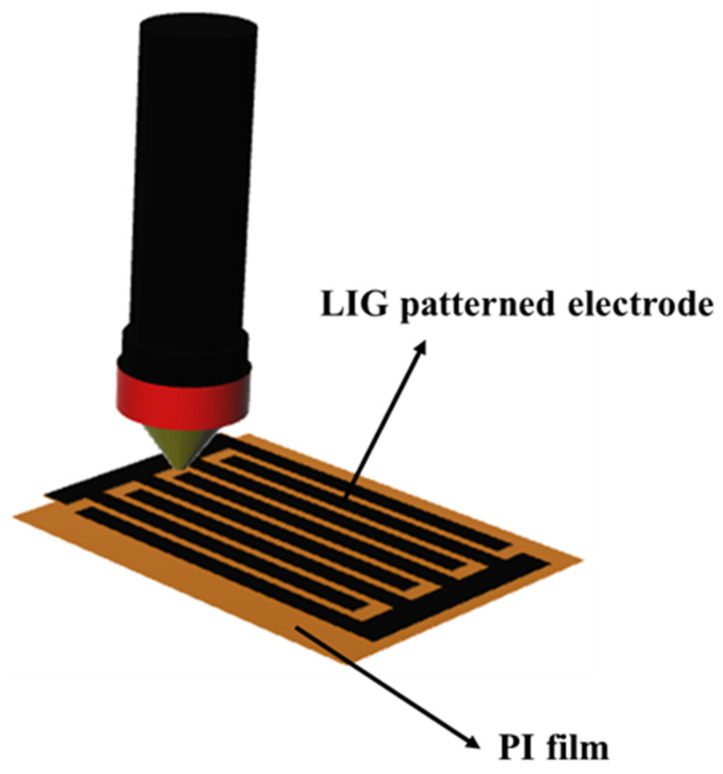
Schematic of the fabrication method of LIG array on PI film.

Using a CO_2_ laser engraving system in a normal air environment, a CO_2_ laser was continuously irradiated on the specific area of the PI film. The array of electrodes on the PI film was designed using computer-aided design (CAD). Scanning power of irradiation of 6 W was used and the velocity of scanning was 450 mm s^−1^. The gap between scan steps was 0.025 mm. The typical process time of CO_2_ laser irradiation was approximately 7 min for an LIG film having a size of 50 × 50 mm^2^. A schematic of the fabrication of the LIG is shown in Figure 1.

### 2.2. Preparation of Circular Padded Film of Silicone Rubber

**Figure 2:**
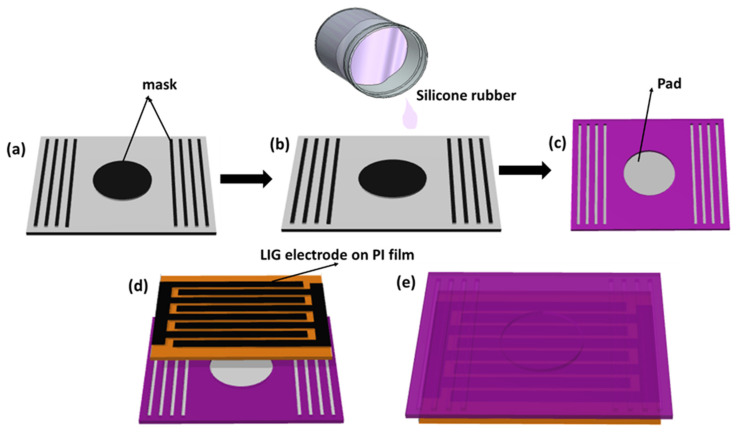
The schematic of the fabrication of ATPS. (**a**) The mask placed on the thick paper to create a pad and some frictional layers. (**b**) Pouring of silicone rubber onto the masked paper. (**c**) Padded silicone rubber film. (**d**) LIG electrode with a PI film and a film of padded silicone rubber vertically attached. (**e**) Schematic of the air-locked ATPS in working condition.

A thick paper was taken and the upper surface was covered using double-sided tape. A circular aluminum plate with a 0.6 mm thickness and a 1.2 cm diameter was placed in the middle of the paper. At each side of the circular plate, at a distance of 5 mm, four straight wires, 1 mm in diameter and 1.2 cm long, were placed at a distance of 2 mm from each other (Figure 2a). Part A and part B of the silicone rubber were taken in a 1:1 mass ratio and stirred; finally, the mold was drop-cast on the mask-designed thick paper (Figure 2b). Finally, after 1 h, the dried silicone rubber film was removed from the paper and, hence, we obtained a padded film with a friction enhancing layer, as shown in Figure 2c.

### 2.3. Characterization and Electrical Output Measurements

Field emission scanning electron microscopy (FESEM) and colored energy-dispersive X-ray spectroscopy (EDS) images of an intrinsic silicone foam were taken using an FEI device (Apreo S HiVac, Thermo Fisher Scientific, Waltham, MA, USA), and Fourier transform infrared spectrometry (FT-IR) analysis was performed using a Bruker Optik GmbH device (Vertex-70V/Hyperion 3000, Keyence, Tampa, FL, USA).

The electrical output signal was measured using a digital oscilloscope (TBS 2202 B, Tektronix, Beaverton, OR, USA), a high-voltage probe (P5100 A, Tektronix, USA) and a current amplifier (DLPCA-200, Femto, Berlin, Germany).

### 2.4. Fabrication of Air-Trapped Padded Sensor (ATPS)

The padded films of silicone rubber and LIG were vertically attached together using tape in such a way that no air leakage took place, as shown in Figure 2d,e. The resultant thickness of the ATPS was 1 mm.

## 3. Results and Discussion

A number of approaches to enhancing the output voltage of TENGs have been devised to date, using popular tribo-negative polymers such as polydimethylsiloxane (PDMS), polyvinylidene fluoride (PVDF), nylon and silicone rubber [2,9,33,34,35]. Various triboelectric sensors focusing on the use of dielectric materials have been reported to date [36,37]. In our study, we utilized air, which possesses a dielectric constant of 1, as an enhancer of the triboelectric output. The ultrathin and ultrasensitive triboelectric sensor, the ATPS, was fabricated using LIG and SR. A schematic of the fabrication of the LIG film is shown in Figure 1, and the fabrication process is described in the Materials and Methods. The thickness of the LIG was 0.25 mm. Our ATPS is a single-electrode system, with the only electrical connection being to the LIG.

**Figure 3:**
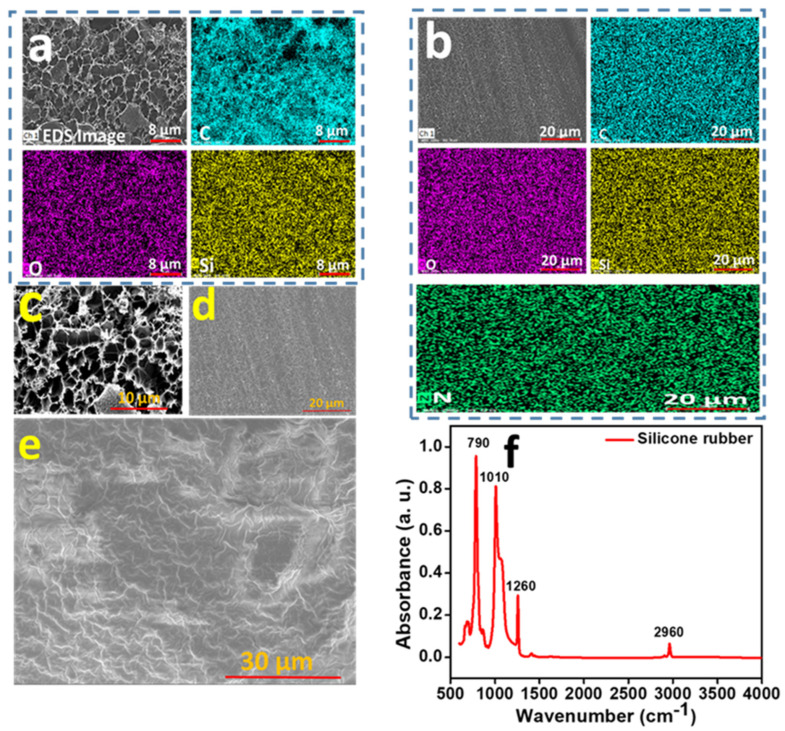
(**a**) EDS of silicone rubber. (**b**) EDS of PI film. (**c**) SEM of LIG. (**d**) SEM of PI film. (**e**) SEM of the film of silicone rubber. (**f**) FTIR spectra for silicone rubber.

The trapping of air was accomplished by utilizing a pad created with SR. The scheme of the fabrication of the pad is described in the Materials and Methods section, with corresponding figures provided in Figure 2a–c. The vertical attachment between the PI and SR is depicted in Figure 2d. The trapping was achieved by using a silicone rubber pad and PI film. During the fabrication of the device, we took care to ensure that there was no air leakage in the ATPS, as shown in Figure 2e. The characterization for SR was conducted through SEM, FTIR and color mapping energy-dispersive X-ray spectroscopy (EDS). For the PI film, color mapping EDS and SEM were performed. To observe the nature of the surfaces of the grown LIG on the PI film, we took SEM images of the LIG. We characterized the SR and PI films by EDS, as shown in Figure 2a,b, respectively. We can observe from the EDS results that the silicone rubber contained the elements carbon (C), oxygen (O) and silicon (Si), as shown in Figure 3a. From the FTIR spectra, we can see the presence of hydrogen-associated bonds, indicating the presence of hydrogen (H) in the SR. However, it is important to note that hydrogen was not detectable in EDS. On the other hand, the PI contained nitrogen (N), carbon (C), oxygen (O), silicon (Si) and hydrogen (H), as shown in Figure 3b. Notably, hydrogen was not detected by EDS. The surface morphology of the LIG, PI and SR was examined using scanning electron microscopy (SEM), as shown in Figure 3c–e, respectively. 

We can observe that the electrode grown under laser irradiation on PI is porous and exhibits a continuous nature, as shown in Figure 3c. The SEM images reveal that the surface of the SR is rougher than surface of the PI, which is desirable in TENGs as it helps to achieve higher friction when they come into contact with each other, as shown in Figure 3d,e. We conducted Fourier transform infrared spectroscopy (FTIR) analysis on the SR to identify the functional groups present. The characteristic peaks in the FTIR spectrum reveal the presence of all bonds required to form silicone rubber, as illustrated in Figure 3f. The characteristic peak at 790 cm^−1^ corresponds to the presence of the Si-(CH_3_)_2_ group, the peak at 1010 cm^−1^ corresponds to the main chain of the Si-O-Si bond, the peak at 1260 cm^−1^ corresponds to the Si-CH_3_ group, and the peak at 2960 cm^−1^ corresponds to the vibrations of the C-H bond in the CH_3_ group [38]. 

**Figure 4:**
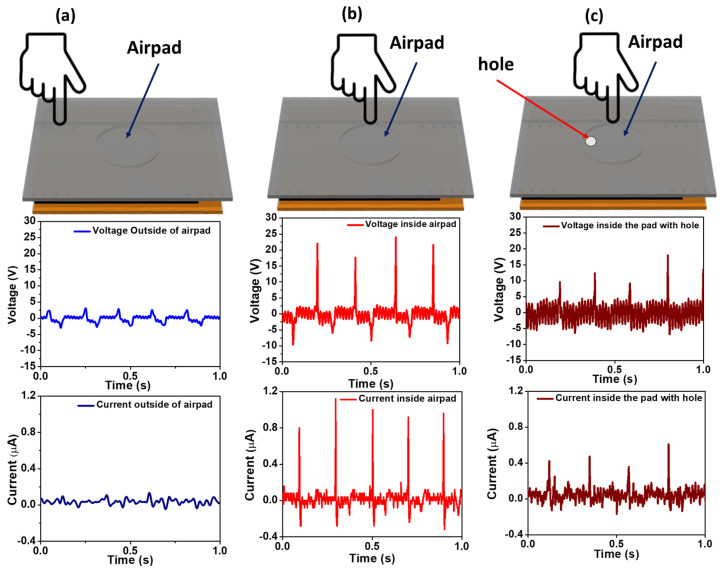
Output voltage and current while tapping. (**a**) Outside the trapped air pad. (**b**) On the trapped air pad. (**c**) On the air pad with a hole.

The electrical output characteristics were determined by applying force through tapping the air-trapped pad with a finger. The sensor was a single-electrode circuitry system, where the only connection was to the LIG electrode, and effective triboelectrification took place in the air-trapped pad. The single-electrode system makes it less complex, lightweight and user-friendly, enabling the fabrication of a leakage-free sensor. Frictional layers on both sides of the air pad were developed, capable of participating in the triboelectrification process when the device undergoes twisting or stretching, as indicated by the black lines in Figure 2a,b. The combination of the air pad and frictional layers on both sides makes the sensor highly responsive to various externally applied forces generated by different body parts. Therefore, the ATPS shows potential application in the field of healthcare sensing. Except for the air-trapped pad, which has a gap of 0.6 mm, the other parts of the silicone rubber film are fully attached to the PI film. This ensures that the ATPS is fully airtight. To verify the uniqueness of the trapped-air pad and the frictional layers, we compared the output from the three devices, as shown in Figure 4a–c. We applied external tapping both on the outside and inside of the pad, measured the voltage and current and compared the effectiveness of the trapped air inside the pad. We applied an external force to the outside of the pad, as shown in Figure 4a. A very low output voltage of 6 V and a current of 0.12 µA resulted from the finger effect on the SR film. Since there is no gap between the SR and PI film, no contact–separation occurs between the PI and SR film, as shown in Figure 4a. When tapping is applied to the air-trapped pad, the output voltage dramatically increases fivefold to 32 V, and the current increases tenfold to 1.2 µA, as shown in Figure 4b. The increase in output voltage is attributed to the contact–separation and the trapped charged air in the pad. Due to triboelectrification, the trapped air becomes charged simultaneously with the generation of static charges on the surfaces of the PI and SR films, contributing to the higher electrical output. The effect of charged air was validated by creating a hole in the pad and measuring the output voltage and current, as shown in Figure 4c. The hole in the pad allows air to pass from the inside to the outside during contact–separation, resulting in a significant drop in the output voltage and current, with average values of 20 V and 0.8 µA, respectively. This suggests that the charged and trapped air helps to enhance the electrical output in our device. By utilizing trapped air as a dielectric medium, we fabricated the ATPS, which generates a higher current of 1.2 µA, corresponding to the current density of 10.6 mA·m^−2^. Therefore, we observed ultra-sensitive output characteristics in the sensing application. 

**Figure 5:**
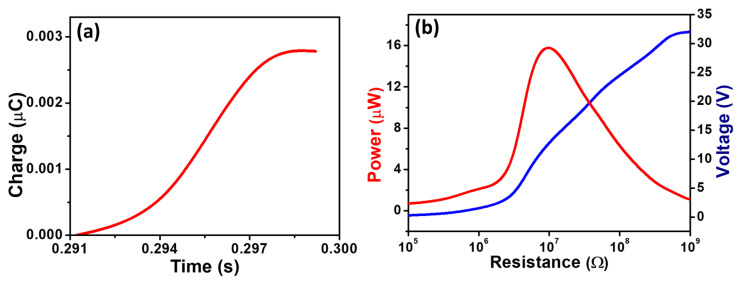
(**a**) Plot for amount of transferred charge at each pressing time. (**b**) The output power and the variation in the output voltage under different levels of external resistance.

We studied the electrical characteristics of the ATPS exclusively using the air-trapped padded device, as it provided a higher electrical output compared to the pad with a hole, as shown in Figure 4b,c. However, we also measured the amount of transferred charge at each pressing time, as shown in Figure 5a. By integrating a positive peak of the current pulse, we can observe that the transferred charge for each strike is approximately 0.0027 µC, corresponding to a charge density of 23.9 µC m^−2^. 

**Figure 6:**
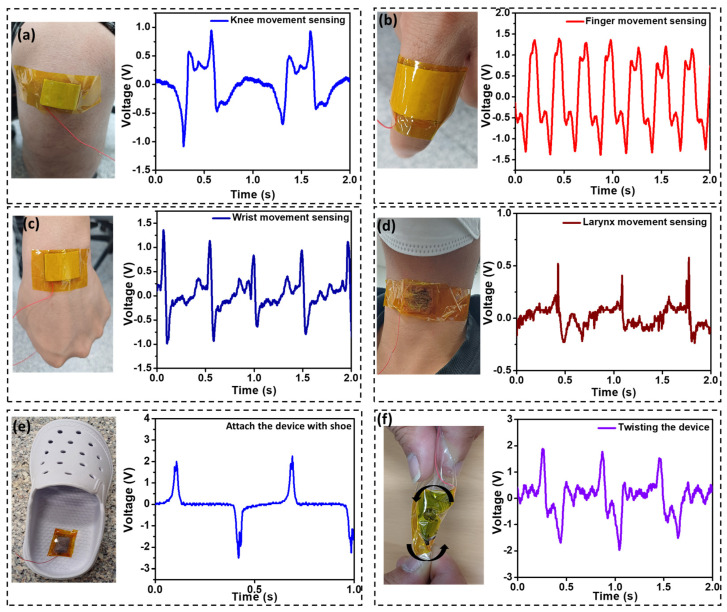
Multipurpose applications of ATPS. (**a**) Knee movement sensing. (**b**) Finger movement sensing. (**c**) Wrist movement sensing. (**d**) Larynx movement sensing. (**e**) Step recording. (**f**) Voltage corresponding to twisting of the device, where the arrow shows the direction of the twist.

Similarly, when measuring the output voltage across the external resistance, we observed that the output voltage changed linearly with the resistance. It increased with the increase in the value of resistance and became saturated at 32 V when the resistance reached 1GΩ, as shown in Figure 5b. The high value of 1GΩ mimics an open-circuit voltage, resulting in an output voltage of 32 V, similar to the open-circuit output voltage shown in Figure 4b. It is necessary to measure the output power of a device under its working conditions. The output power of the ATPS was checked, and we obtained a maximum of 15.8 µW at a resistance value of 10 MΩ, as shown in Figure 5b. Therefore, we achieved the maximum power density of 139.8 mW m^−2^ at a resistance level of 10 MΩ. At this level of resistance, the device becomes highly electrically sensitive, as it can deliver higher output even under very low amplitudes of external force. Our ATPS is highly sensitive to a versatile range of low-pressure amplitudes, and we have demonstrated its potential application in healthcare sensing. To make our device compatible for real-time applications in the field of healthcare sensing, we sought to develop a highly flexible, twistable and robust sensor. To achieve this, we replaced the LIG with conductive carbon tape without compromising the air-trapped pad. The conducting carbon tape serves as both the triboelectric material and the conducting electrode. We attached carbon tape with dimensions of 20 × 15 mm^2^ to the padded film of SR, ensuring no air leakage. The only electrode was connected to the conductive carbon tape. The resultant device was applied in multipurpose healthcare sensing applications, as depicted in Figure 6.

We have provided a table comparing the output performance of triboelectric sensors reported previously with that developed in our research work, as shown in Table 1. This provides an insight into the potential real-time application of our sensor, the ATPS [39,40,41,42,43,44,45,46]. 

We attached our sensor to the knee, and, with each small movement, the sensor recorded the corresponding signal. When the knee is folded, the SR and carbon tape come into contact, resulting in a positive peak of 0.95 V. Similarly, when the leg straightens, separation occurs between the films, producing a negative pulse of 0.45 V, as shown in Figure 6a. This indicates that our sensor can be utilized to monitor the up–down cycle during knee exercises based on the peak count. The position of the ATPS’ attachment is crucial; the most intense peaks were obtained when the device was attached at the knee joint. The use of other positions on the knee may disrupt the efficiency of the ATPS, as proper contact–separation may not occur, leading to discontinuous peak counts for each folding and straightening movement of the leg. In the case of conscious patients recovering from an illness, finger movement is often one of the first signs of recovery. For this reason, we attached our sensor to the middle portion of a finger, as shown in Figure 6b. Here, when the finger is folded down, contact occurs between the SR film and the carbon conductive tape, resulting in a positive peak with a value of 1.55 V. Similarly, when the finger is in a straight position, the films separate, and a corresponding negative peak of 1.35 V is observed; see Figure 6b. We also registered wrist movement by attaching the sensor to the wrist of a hand, as shown in Figure 6c. A straight-down or angled move causes contact, while an upward move causes separation between the two films. Each action generates a positive and negative voltage peak, with values of 1.1 V and 0.9 V, respectively. Optimal positioning at the wrist junction enhances the signal output by improving the contact and separation. The larynx is a vital organ for speech and breathing cycles [47]. Patient recovery often involves attempting to talk, causing the larynx to move or vibrate. To capture this movement, we attached our device to the larynx, as shown in Figure 6d. During speech, the larynx movement results in contact–separation between the two films in the pad, producing positive and negative peaks of 0.52 V and 0.25 V, respectively. These signals were recorded in the output. The sensing of larynx movement is a crucial application in the field of triboelectric sensors. In modern healthcare monitoring, physical exercise plays a significant role. Gait movement is particularly important, as various forms of walking and running have positive impacts on the human body. To incorporate our ATPS device into gait sensing, we attached the sensor to a shoe. With each press and release of the heel, the device registered positive and negative peaks with values of 2 V and 2.5 V, respectively; see Figure 6e. The device enables step counting, making it useful for health monitoring during running or walking. Finally, we tested the robustness of the device by twisting it, as shown in Figure 6f. Changes in the direction of the twist can generate corresponding positive and negative voltage peaks. In each application, optimal sensing results were observed when the device was properly positioned on the corresponding body parts, allowing for full contact–separation between the two films. While we demonstrated the electrical characteristics of the ATPS by manually applying force through tapping the air pad, the stability of the sensor over a large number of cycles was not explored. However, since silicone rubber and PI are highly stable in ambient conditions for prolonged periods, we anticipate that the ATPS will deliver a stable output over an extended duration. The significance of the trapped air in the pad, leading to a higher electric output, and the broad range of applications in healthcare sensing make the ATPS crucial for real-time biomedical applications.

## 4. Conclusions

We fabricated a triboelectric sensor using an air-trapped pad with LIG. The novelty of the device fabrication, utilizing air as a dielectric layer, offers opportunities for research in the field of triboelectric nanogenerators. In our work, we demonstrated the efficacy of trapped air as an effective dielectric layer by comparing it with a normal padded device that had a hole. We highlighted the difference in output between the two devices. The ATPS generated an output voltage of 32 V and a power density of 139.8 mW m^−2^. With a device size of 20 × 15 × 1 mm^3^ and effective padding area spacing of only 0.6 mm, it can be utilized in a wide range of pressure sensing applications. LIG plays a crucial role in keeping the device thin, as the array of electrodes was grown on the surface of a PI film using CO_2_ laser irradiation. The significance of the charged air, its role in creating a highly sensitive sensor and the versatility in healthcare sensing applications make our device attractive for potential applications in the field of triboelectric nanogenerators. 

## Figures and Tables

**Table 1:** A comparative study of reported healthcare sensors with our device, the ATPS.

Ref. Work	Type	Materials Used	Size	Mode of Operation	Max. Current	Power Density
[39]	Sleep sensor	APLF–Cu	1.8 cm^2^	Contact–separation	0.9 µA	120 mW/m^2^
[40]	Motion sensor	ABS–Cu	-	Contact–separation	0.26 µA	0.22 µW/cm^3^
[41]	Cardiovascular sensor	FEP–PET	1.5 × 3 cm^2^	Contact–separation	-	23 μW/m^2^
[42]	Coronary heart disease sensor	Kapton–Cu	20 × 10 mm^2^	Contact–separation	2.73 µA	-
[43]	Tactile sensor	Ionogel–PDMS	2 × 1.5 cm^2^	Contact–separation	2.3 nA	-
[44]	Touch sensor	P(VDF-TrFE)–PDMS	-	Contact–separation	-	46.7 µW/cm^2^
[45]	Respiratory sensor	PTFE–Al	3 × 2 × 0.1 cm^3^	Contact–separation	4 µA	-
[46]	Pulse sensor	PET	40 × 20 mm^2^	Single-electrode mode	5.4 nA	-
**Our work**	**Multipurpose pressure sensor**	**LIG–silicone rubber**	**1.13 cm^2^**	**Contact–separation**	**1.2 µA**	**139.8 mW/m^2^**

## Data Availability

The data are available on reasonable request from the corresponding author.
